# Acute kidney injury in infants with congenital diaphragmatic hernia

**DOI:** 10.1038/s41372-022-01378-6

**Published:** 2022-04-07

**Authors:** Fahad M. S. Arattu Thodika, Theodore Dassios, Akash Deep, Anne Greenough

**Affiliations:** 1 Department of Women and Children’s Health, School of Life Course Sciences, Faculty of Life Sciences and Medicine, King’s College London, London, UK; 2 Neonatal Intensive Care Centre, King’s College Hospital NHS Foundation Trust, London, UK; 3 Paediatric Intensive Care Unit, King’s College Hospital NHS Foundation Trust, London, UK; 4 Asthma UK Centre for Allergic Mechanisms, King’s College London, London, UK; 5 NIHR Biomedical Research Centre based at Guy’s and St Thomas’ NHS Foundation Trust and King’s College London, London, UK

**Keywords:** Kidney, Kidney diseases

## Abstract

**Objectives:**

To assess the incidence of acute kidney injury (AKI) in infants with congenital diaphragmatic hernia (CDH), including those who had fetoscopic endoluminal tracheal occlusion (FETO), and the effect of AKI on mortality and length of stay.

**Study design:**

Ten-year retrospective review of infants admitted with CDH to a tertiary perinatal centre.

**Result:**

Ninety-four infants with median gestational age of 38^+1^ weeks were included. Fifty-nine (62.8%) infants had AKI. Compared to infants without AKI, infants with AKI, had a similar incidence of mortality (*p* = 0.989). In survivors, AKI was not independently associated with a longer adjusted median length of stay [23 versus 15 days (*p* = 0.194)]. FETO was associated with an increased risk of AKI (*p* = 0.005), but neither the mortality nor length of stay of FETO infants who had AKI was increased.

**Conclusion:**

AKI was present in the majority of infants with CDH and most common in those who had undergone FETO.

## Introduction

Acute kidney injury (AKI) occurs in 30% of critically ill neonates and is associated with increased mortality and prolonged hospital stay [1]. AKI in the newborn period has been underdiagnosed, as it can be thought to reflect maternal creatinine values [2]. The modified neonatal acute kidney definition by the organisation “Kidney Disease: Improving Global Outcome” (KDIGO) has made it easier to identify the affected neonatal population (Table 1) [2, 3]. The Assessment of Worldwide Acute Kidney Epidemiology in Neonates study was a large multicentre, observational study which validated the definition of AKI in neonates and reported the incidence of AKI and associated morbidities [1, 2].

**Table 1:** Staging and definition of neonatal acute kidney injury (KDIGO).

Stage	Serum creatinine (SCr)
**0**	No change in SCr or rise < 0.3 mg/dl
**1**	SCr rise ≥ 0.3 mg/dl within 48 h or SCr rise ≥ 1.5 to 1.9 X reference SCr^a^ within 7 days
**2**	SCr rise ≥ 2 to 2.9 X reference SCr^a^
**3**	SCr rise ≥ 3 X reference SCr^a^ or SCr ≥ 2.5 mg/dl or Receipt of dialysis

^a^Reference SCr is the lowest prior SCr measurement.

Congenital diaphragmatic hernia (CDH) is a rare congenital anomaly characterised by herniation of visceral contents into the thoracic cavity [4]. Affected infants are often critically ill with hypoxic respiratory failure requiring high levels of ventilatory support, inotrope administration and nephrotoxic drugs that could lead to acute kidney injury. Previously reported studies have emphasised the significance of AKI in CDH infants and its association with mortality, duration of invasive ventilation and length of neonatal stay [5, 6]. Some of those infants required extracorporeal membrane oxygenation (ECMO) which per se can either lead to AKI or aggravate pre-existing AKI [7]. The need for ECMO combined with AKI in CDH infants is associated with increased mortality, duration of ECMO support and ventilation days [8, 9].

Our aims were to identify the burden of AKI in infants with CDH in a tertiary neonatal surgical centre, where a high proportion of infants had severe CDH and hence had had an antenatal intervention, fetoscopic endoluminal tracheal occlusion (FETO). To our knowledge, the effect of FETO in CDH infants on the occurrence of neonatal AKI and outcomes in CDH has not been previously described. We explored the effect of AKI on mortality and length of stay and whether these outcomes differed between those with severe AKI versus mild or no AKI. We also identified the effect of AKI on mechanical ventilation and intensive care free days. Further analysis was undertaken to determine whether AKI was commoner in infants who underwent FETO and if it influenced their outcomes.

## Subjects and methods

This was a retrospective, observational study conducted in the perinatal centre at King’s College Hospital NHS Foundation Trust (KCH), London, UK. Infants admitted with CDH between 01/01/2011 to 01/01/2021 were included in the study. Infants with associated renal anomalies were excluded. The study was registered with King’s College Hospital NHS Foundation Trust Clinical Governance and Audit Department.

KCH is a tertiary neonatal surgical centre which offers antenatal intervention for infants with CDH. Infants with moderate to severe left sided CDH were randomised either to receive expectant management or undergo FETO as part of the TOTAL trial (NCT01240057) from 2013. Prior to 2013, parents of infants with either left or right sided CDH were given the choice of FETO for their foetus on compassionate grounds [10]. Infants were managed based on the CDH EURO Consortium guidelines [11]. The majority of infants were ventilated from birth and were given sedation (morphine or fentanyl infusions) and muscle relaxing agents in severe cases. Infants often had pulmonary hypertension necessitating inhaled nitric oxide and inotrope administration. All infants were started on broad spectrum antibiotics (benzyl-penicillin and gentamicin) which were changed to cephalosporins, vancomycin and beta lactams in cases of clinically suspected or proven secondary infection.

In the UK there are six centres specialised to provide respiratory ECMO. Infants who require ECMO for various respiratory pathologies including CDH are referred to those centres via the Children’s Acute Transport Service (CATS). CDH infants with a birthweight more than 2000 grams and gestational age more than 34 weeks of gestation with oxygenation index more than 25 despite maximal conventional support are considered for ECMO. Infants from our perinatal centre who require ECMO are referred to one of these centres via CATS [12].

Neonatal AKI was defined and classified based on the KDIGO definition (Table 1) into stage 0, 1, 2 and 3 using reference serum creatinine values [2]. Serial serum creatinine values were extracted from the electronic patient records and infants with at least two serum creatinine values were included in the study. The lowest serum creatinine value prior to the rise in creatinine value was considered as the reference serum creatinine value. Urine output was not used to diagnose AKI due to the lack of consistency in documentation of urine output. The highest stage of neonatal AKI and the day of occurrence of the same were noted. Previous studies have shown that stage 1 AKI could be transient and morbidity and mortality is more likely associated with severity of AKI in paediatric population [13, 14]. Henceforth, these infants were further classified into those with severe AKI (stage 2 and 3) and no (stage 0) or mild (stage1) AKI to identify if that affected the outcomes.

Demographic data were collected from the medical records and included—prenatal diagnosis, observed to expected lung head ratio (O/E LHR) at diagnosis, antenatal FETO, side of defect, presence of liver in thorax confirmed intraoperatively, gestational age, birthweight, gender, mode of delivery, Apgar score at 5 min, male gender, use of inhaled nitric oxide, inotrope administration, highest oxygenation index (OI), sepsis (defined as positive blood culture), day of surgery, patch repair and use of nephrotoxic medications. The postnatal characteristics included were the presence of neonatal AKI, stage of neonatal AKI, severity of AKI, need for ECMO, duration of invasive ventilation, mortality, ventilator free days by day 28 of life, intensive care unit free days by day 28 of life and length of neonatal stay (survivors) [15]. In our unit, infants with suspected sepsis are started on benzyl penicillin and gentamicin as first line antibiotics. These antibiotics are escalated to cefotaxime and vancomycin if the infant is either not responding to first line after 48 h or there is biochemical evidence of increasing C reactive protein. Beta lactam antibiotics such as meropenem are reserved for infants not responding to second line antibiotics.

### Analysis

The data were tested for normality using Kolmogorov-Smirnov’s test and found to be non-normally distributed, hence, the risk factors and outcomes associated with AKI were assessed for statistical significance using the Mann-Whitney U test for continuous variables and the Chi-square test for dichotomous variables. The relationship of length of stay in the survivors with gestational age, birthweight, Apgar at 5 min and LHR at diagnosis were calculated using Kendall-tau correlation coefficient. Factors significantly related to the length of stay (*p* < 0.05) were inserted in a linear regression model to identify their independent association with the length of stay. The effect of severe AKI versus mild or no AKI on mortality and length of stay were also performed. Further analysis was undertaken to determine if AKI was more common in infants who underwent FETO and in that group did it effect their outcomes. IBM SPSS Statistics for Windows, Version 27.0 (SPSS Inc. Chicago, IL) was used to analyse the data.

## Results

One hundred and five infants with CDH were admitted during the study period. Eleven of the infants were excluded, one infant had an associated renal anomaly (polycystic kidney disease) and ten infants died within the first 12 h after birth and had only one creatinine value. The 94 infants included in the study had a median gestational age of 38^+1^ (range: 25^+3^–41^+5^) weeks and a birthweight of 2710 (750–3335) g. Thirty-two infants (37.6%) had had FETO, their median observed to expected LHR at diagnosis was 32.0 (range: 11.0–51.8) %. Overall, 59 (62.8%) infants survived to discharge. Five infants were transferred for ECMO, three survived to discharge.

Fifty-nine (62.8%) infants had neonatal AKI; 37 (62.7%) had stage 1, 18 (30.5%) had stage 2 and 4 (6.8%) had stage 3 neonatal AKI. The median (IQR) onset of AKI was 2 (1–4) days. In survivors, the AKI resolved within 72–96 h. Four infants had stage three AKI, none of these infants underwent CRRT. One of the infants died and another infant was too sick to undergo the procedure at that point of time.

A lower median O/E LHR at diagnosis was associated with a higher risk of developing AKI (28.2 versus 36.9%, *p* = 0.021). Furthermore, CDH infants with AKI had a higher oxygenation index [32.9 (15.3–69.5)] compared to infants without AKI [16.5 (5.2–64.3), *p* = 0.027] (Table 2). The occurrence of AKI was similar in infants who required ECMO (5%) compared to infants who did not require ECMO (5%, *p* = 0.89) (Table 2).

**Table 2:** Demographics according to severity of AKI.

Antenatal characteristics	Neonatal AKI		*p* Value	Severe neonatal AKI		*p* value
	Yes (*N* = 59)	No (*N* = 35)		Yes (*N* = 22)	No (*N* = 72)	
Antenatally confirmed	52 (88.1)	32 (91.4)	0.617	19 (86.4)	65 (90.3)	0.602
FETO^a^	26 (49.1)	6 (18.8)	0.005	11 (57.9)	21 (31.8)	0.039
Observed to expected LHR at diagnosis^a^	28.2 (21.2–38.7)	36.9 (30.5–42.8)	0.021	31.0 (24.0–40.0)	33.0 (24.3–43.5)	0.835
Right sided CDH	8 (13.6)	6 (14.3)	0.921	3 (13.6)	10 (13.9)	0.976
Liver in thorax	8 (13.6)	6 (14.3)	0.921	3 (13.6)	10 (13.9)	0.976
*Postnatal characteristics*						
Gestational age (weeks)	38.1(34.7 –39.1)	38.1 (34.1–39.1)	0.925	38.1 (34.3–38.8)	38.1 (34.9–39.1)	0.483
Birthweight (kg)	2.72 (2.25–3.18)	2.7 (1.97–3.13)	0.684	2.69 (2.27–3.17)	2.74 (2.2–3.16)	0.837
Male Gender	37 (62.7)	17 (48.6)	0.18	14 (63.6)	40 (55.6)	0.502
Caesarean	22 (37.9)	11 (31.4)	0.561	7 (31.8)	26 (36.1)	0.71
Apgar at 5 min	7 (6–9)	8 (7–10)	0.10	7 (7–9)	7 (6 –9)	0.350
Inhaled Nitric oxide	42 (71.2)	19 (54.3)	0.097	14 (63.6)	47 (65.3)	0.88
Inotropes	55 (93.2)	28 (80)	0.054	21 (95.5)	62 (86.1)	0.23
Highest Oxygenation index	32.9 (15.3–69.5)	16.5 (5.2–64.3)	0.027	54.9 (23.3–74.4)	19.1 (7.8–53.1)	0.007
Culture proven sepsis	11 (18.6)	2 (5.7)	0.079	3 (13.6)	10 (13.9)	0.98
Day of Surgery	4 (4–6)	4 (3–5)	0.164	6 (3–8)	4 (3–6)	0.67
Patch repair	18 (42.9)	6 (27.3)	0.221	8 (50)	16 (33.3)	0.23
ECMO use	3 (5.1)	2 (5.7)	0.89	1 (4.5)	4 (5.6)	0.853

Data expressed as median (IQR) or *n* (%).

^a^Only in prenatally diagnosed cases = 84.

The use of vancomycin was associated with a higher incidence of AKI (57.6% versus 33.3%; *p* = 0.03). Meropenem administration was also associated with a higher incidence of AKI (28.8 versus 2.9; *p* = 0.002) (Table 3).

**Table 3:** Nephrotoxic medications used in NICU.

Nephrotoxic drugs	Neonatal AKI		*p* Value
	Yes (*N* = 59)	No (*N* = 35)	
Gentamicin	59 (100)	35 (100)	-
Vancomycin	34 (57.6)	11 (33.3)	0.025
Meropenem	17 (28.8)	1 (2.9)	0.002
Inhaled Nitric oxide	42 (71.2)	19 (54.3)	0.097
Inotropes	55 (93.2)	28 (80)	0.054
Diuretics	12 (20.3)	3 (8.6)	0.132

Data expressed as *n* (%).

The mortality was similar in infants with AKI (37.3%) compared to infants without AKI (37.1%) *p* = 0.989) (Table 4). In the survivors, AKI was associated with a longer median length of stay (23 versus 15 days, *p* = 0.003) (Table 4), but regression analysis identified that neonatal AKI was not independently associated with the length of stay (adjusted *p* = 0.194).

**Table 4:** Outcomes according to severity of AKI.

Outcomes	Neonatal AKI		*P* value	Severe neonatal AKI		*p* Value
	Yes (*N* = 59)	No (*N* = 35)		Yes (*N* = 22)	No (*N* = 72)	
Duration of invasive ventilation^a^ (days) (*N* = 59)	10 (8–16)	6 (5–8)	0.06	11 (6–21)	8 (6–12)	0.181
Length of neonatal stay^a^ (days) (*N* = 59)	23 (8–47)	15 (1–20)	0.003 adjusted p = 0.194	29 (23–54)	22 (17–40)	0.198
Ventilation free days (days)	7 (0–20)	19 (0–22)	0.131	9 (0–19)	12 (0–21)	0.90
Intensive-care unit free days (days)	2 (0–18)	11 (0–20)	0.248	3 (0–19)	7 (0–19)	0.937
Mortality	22 (37.3)	13 (37.1)	0.989	7 (31.8)	28 (38.9)	0.548

Data expressed as median (IQR) or *n* (%).

^a^In infants who survived = 5.9.

The number of ventilator-free days were similar in CDH infants with AKI [7 (0–20)] to CDH infants without AKI [19 (0–22); *p* = 0.13]. Intensive care free days were also similar in infants with AKI [2 (0–18)] to infants without AKI [11 (0–20); *p* = 0.25] (Table 4).

A further analysis was undertaken to compare if severe AKI was associated with mortality and length of stay. Twenty-two (23.4%) of the 94 included infants had severe AKI. The mortality was similar in infants with severe AKI (31.8%) and those with no or mild AKI [(38.9%), *p* = 0.548]. The length of stay in survivors was similar in infants with severe AKI [29 (23–54) days] compared to infants with no or mild AKI [23 (17–40) days; *p* = 0.198]. Furthermore, the ventilator free days were similar in infants with severe AKI [9 (0–19)] and those with no or mild AKI [12 (0–21); *p* = 0.90]. The ICU free days were also similar in infants with severe AKI [3 (0–19)] compared to infants with no or mild AKI [7 (0–19); *p* = 0.94] (Table 4].

Infants who underwent FETO had an increased incidence of AKI compared to those who did not have FETO (49.1% versus 18.8%, *p* = 0.005. Amongst the 26 FETO infants who had AKI, 15 (58%) had stage 1, 9 (35%) had stage 2 and 2 (7%) had stage 3. Mortality did not differ significantly between FETO infants with AKI (50%) and those without AKI (50%; *p* = 1.000). Furthermore, the presence of AKI did not significantly increase the length of hospitalisation in surviving infants with AKI (54 days) compared to infants without AKI (40 days, *p* = 0.521) (Table 5).

**Table 5:** AKI in infants who had FETO.

Characteristics	FETO [*N* = 32]		
	AKI [*N* = 26]	No AKI [*N* = 6]	*p* value
O/E LHR at diagnosis	26.5 (17.5–35.0)	35.2 (22.5–37.5)	0.374
Right sided CDH	5 (19.2)	0	0.242
Gestational Age (weeks)	34.6 (33.9–35.7)	33.4 (31.3–36.2)	0.308
Birthweight (kg)	2.26 (2.0–2.68)	2.01 (1.58–2.64)	0.524
Apgar at 5 min	7 (6–8)	6 (3–7)	0.308
Duration of invasive ventilation^a^ (days)	21 (16–23)	10 (9–11)	0.237
Length of stay^a^ (days)	54 (36–82)	40 (31–59)	0.521
Ventilation free days	1 (0–8)	8.5 (0–18)	0.436
Intensive care free days	0 (0–2)	1 (0–11)	0.796
Mortality	13 (50)	3 (50)	1.000

Data expressed as median (IQR) or n (%).

^a^In infants who survived = 16.

## Discussion

We have demonstrated that AKI occurs in the majority of infants with CDH and that FETO was associated with an increased risk of neonatal AKI. The use of higher spectrum antimicrobials and a higher OI increased the risk of neonatal AKI in infants with CDH. We also report that in a population of infants with CDH including those require FETO, AKI was not an independent predictor of survival or duration of hospitalisation. Neither did AKI independently affect the ventilator or the intensive care free days.

In our study, neonatal AKI was diagnosed in approximately 63% of the population, whereas previous studies have reported the incidence varies from 38 to 71% [5, 6, 8, 9]. The increased incidence is likely because of the severe nature of the CDH in our cohort. This is supported by the findings that infants in our cohort with AKI had a median (IQR) O/E LHR at diagnosis of 28.2 (21.2–38.7) compared to the infants in a previous study whose O/E LHR was 38.5 (24.9–48.0) [5]. In addition to this our results are similar to findings of the CDH subgroup in two previous studies that looked into the incidence of AKI in infants who underwent ECMO [8, 9].

Our study found that ionotrope administration, sepsis, day of surgery or patch repair was not associated with increased incidence of AKI. This was similar to findings from the previous studies [5, 8]. Ryan et al. found that inotrope administration and sepsis did not affect the incidence of AKI, but patch repair was associated with an increased incidence of AKI [6].

Our study demonstrated that neonatal AKI was higher in infants who received meropenem or vancomycin. A previously reported study had shown an increased incidence of AKI in CDH infants receiving vancomycin [6]. While previous studies have shown that gentamicin exposure is associated with AKI in infants with CDH [5, 6], our study showed no significant difference. This is may be because all the infants received gentamicin as first line antibiotic in our institution.

The use of ECMO was not associated with neonatal AKI in our population, but only five of our infants underwent ECMO. The increase in incidence of AKI has been previously reported in CDH infants requiring ECMO [5, 8, 9, 16]. In those two studies inhouse ECMO was available [5, 6]. In other studies, CDH infants were included as a subgroup in identifying the incidence of AKI in infants who underwent ECMO for various indications [8, 9, 16].

Our study demonstrated that mortality did not differ significantly in CDH infants with AKI compared to those without AKI. Additionally, AKI was not independently associated with length of neonatal stay in CDH survivors. The ventilator and intensive care unit free days were also similar in CDH infants with AKI compared to those without AKI. A further classification of AKI into two categories—severe AKI and no or mild AKI also showed similar results. Our findings contradict the results of previous studies [5, 6, 8, 9]. This may be explained by the severity of CDH in our population. In our CDH cohort, incidence of AKI was higher in CDH infants with a higher OI value compared to infants with lower OI. Our results imply that the main pathophysiological driver of kidney injury in CDH is probably severe hypoxia and that in the face of global hypoxic respiratory failure, the effect of kidney injury on survival and other specific outcomes is moderated by the overall effect of hypoxic respiratory failure. The finding of a higher incidence of AKI in the FETO group also points toward the same postulate as the infants that were randomised to the antenatal intervention were by definition in the moderate/severe spectrum of the disease as quantified by the observed to expected LHR at diagnosis [17, 18]. The survival in CDH infants is affected by multiple factors including prenatal, postnatal, surgical management. A previously reported study compared the effect of survival in four high volume centres and found it varied significantly between centres [10]. Nonetheless, the CDH survivors who had AKI had to spend on average an extra eight days in the neonatal unit compared to those who did not develop AKI.

The severity of CDH in our cohort was assessed using O/E LHR which has been shown to predict survival [19]. AKI, however, can be affected by postnatal factors such as antibiotic exposure and fluid balance. Furthermore, the number of infants who required ECMO in our cohort was small. This may explain the disparity in the results between our study and previous studies [5, 6, 8, 9].

Our study has strengths and some limitations. This is the largest single centre cohort of CDH infants in whom incidence of AKI was studied and is the first study to report on association of FETO with AKI. Additionally, we defined neonatal AKI using a validated definition by the KDIGO [2]. While one study [5] used the KDIGO, other studies [6, 8, 9] used PRIFLE (Paediatric Risk, Injury, Failure, Loss of function, End stage renal disease) criteria to identify AKI, which is valid in paediatric population rather than neonatal population [20, 21]. Due to the methodological characteristics of our AKI definition, which requires more than one creatinine measurement for diagnosis and staging we, however, had to exclude ten infants with severe hypoxic failure who died very shortly after birth. These infants most likely would have had global hypoxia and AKI, so the true incidence of AKI in our population is likely to be underestimated. Another limitation in the surviving CDH infants who developed AKI was the lack of long term follow up of their renal function post discharge from the neonatal care. Previous studies have shown that neonates with AKI are at risk of developing chronic kidney disease [22, 23]. Annual monitoring for development of hypertension and albuminuria are suggested non-invasive methods of identifying progression to chronic kidney disease in these infants [24].

In conclusion, AKI occurred in the majority of infants in a cohort with severe CDH, and the incidence of AKI was higher in those who required FETO. In infants with CDH, a higher OI was associated with an increased incidence of AKI.

## Data Availability

Data made available on reasonable request.
